# Inter-unit variability of multi-leaf collimator parameters for IMRT and VMAT treatment planning: a multi-institutional survey

**DOI:** 10.1093/jrr/rrz082

**Published:** 2020-01-11

**Authors:** Masaru Isono, Yuichi Akino, Hirokazu Mizuno, Yoshihiro Tanaka, Norihisa Masai, Toshijiro Yamamoto

**Affiliations:** 1 Department of Radiation Oncology, Osaka International Cancer Institute, 3-1-69 Otemae, Chuo-ku, Osaka-shi, Osaka 541-8567, Japan; 2 Oncology Center, Osaka University Hospital, 2-2 (D10), Yamadaoka, Suita, Osaka 565-0871, Japan; 3 Department of Medical Physics and Engineering, Osaka University Graduate School of Medicine, 1-7 Yamadaoka, Suita, Osaka 565-0871, Japan; 4 Department of Radiation Therapy, Japanese Red Cross Society Kyoto Daiichi Hospital, 15-749 Hommachi, Higashiyama-ku, Kyoto-shi, Kyoto 605-0981, Japan; 5 Miyakojima IGRT Clinic, 1-16-22 Miyakojimahondori, Miyakojima-ku, Osaka-shi, Osaka 534-0021, Japan; 6 Department of radiation therapy, Saiseikai Noe Hospital, 1-3-25 Furuichi, Joto-ku, Osaka-shi, Osaka 536-0001, Japan

**Keywords:** MLC leaf transmission, dosimetric leaf gap, IMRT/VMAT commissioning

## Abstract

Modern treatment machines have shown small inter-unit variability regarding beam data. Recently, vendor-provided average beam data, such as the Representative Beam Data (RBD) of the TrueBeam (Varian Medical Systems, Palo Alto, CA, USA), has been used for modeling of the Eclipse (Varian Medical Systems) treatment planning system. However, RBD does not provide multi-leaf collimator (MLC) parameters, such as MLC leaf transmission factor (LTF) and dosimetric leaf gap (DLG). We performed a web-based multi-institutional survey to investigate these parameters as well as the measurement protocols and customization of the parameters for intensity-modulated radiotherapy (IMRT) and/or volumetric modulated radiotherapy (VMAT) commissioning. We collected 69 sets of linear accelerator (linac) data from 58 institutions. In order to measure MLC parameters, most institutions used farmer-type ionization chambers with a sensitive volume of 0.6 cm^3^, water phantoms, source surface distance of 90 cm with 10 cm depth, and a vendor-provided plan. The LTF showed small inter-unit variabilities, although the DLG showed large variations. For optimization of the parameters for IMRT/VMAT calculations, DLG values were upwardly adjusted at many institutions, whereas the LTF values were modestly changed. We clarified that MLC parameters were measured under the same conditions at more than half of the facilities. Most institutions customized parameters in a similar manner for IMRT/VMAT. The median measured and customized values obtained in our study will be valuable to verify MLC installation accuracy and to shorten the iterative processes of finding the optimal values.

## INTRODUCTION

Intensity-modulated radiotherapy (IMRT) and volumetric arc radiotherapy (VMAT) have shown great advantages compared with conventional treatments, in terms of the significant dose reduction delivered to healthy tissues. These technologies also focus increased doses to their targets, leading to improved clinical outcomes in patients [1, 2]. Although IMRT and VMAT achieve excellent dose distributions, accurate modeling of the treatment planning systems (TPSs) and commissioning are necessary in order to conduct accurate beam delivery. Several studies have reported that modern treatment machines showed small inter-unit variability of the beam data due to improved manufacturing [3–7]. A TrueBeam (Varian Medical Systems, Palo Alto, CA, USA) is one of the latest generations of linear accelerators (linacs). For TrueBeam linacs, the vendor provides Representative Beam Data (RBD) [8], which is the mean beam data collected from three TrueBeam machines at one institution, using a CC13 (IBA Dosimetry, Schwarzenbruck, Germany) ionization chamber. Tanaka *et al*. collected beam data of 21 TrueBeam linacs and they reported that the mean beam data was close to the RBD [9]. They also reported very small inter-unit variations among the multiple treatment units. If the RBD is used for beam modeling of the Eclipse (Varian Medical Systems) TPS, the period needed for installation or replacement of the linac can be markedly reduced, although sufficient commissioning is necessary.

The RBD data contains data on percent depth dose (PDD), off-center ratio (OCR) and output factors. For modeling the photon beam data, the Eclipse TPS requires additional data, including the dose monitor unit and multi-leaf collimator (MLC)-related parameters such as the MLC leaf transmission factor (LTF) and dosimetric leaf gap (DLG). The MLC cannot block the photon beams completely and small amounts of radiation is transmited from the leaves. For conventional radiotherapy, such leakage only affects areas outside the treatment field. For IMRT and VMAT, however, MLC leaves often block targets when modulating the photon fluence. Thus, modeling the LTF is important for accurate dose calculation [10, 11]. Usually, the LTF is calculated as the ratio of the measured dose of the open field, defined as the jaws, with and without shielding of the field by MLC leaves. The vendor recommends performing the measurements at several positions and then using average values to ensure the measured value contains both inter-leaf leakage and intra-leaf transmission effects [12]. DLG is a parameter that accounts for the rounded leaf-end transmission effects [13]. When radiation passes through the rounded leaf end, the transmission affects the radiation field edge. In the TPS, this effect is compensated by shifting the leaf tip position by half the value of the DLG when calculating the fluence. Usually the DLG is measured by the sweeping gap procedure described by LoSasso *et al*. [14]. The dose of the sweeping gap leaf motion with various gap widths is measured and the contribution of the leaf transmission is then subtracted. When the values are plotted against the nominal gap width, the DLG value can be obtained by extrapolating the regression line to zero.

Although both LTF and DLG are obtained by measurements, these values are often customized during the commissioning of the IMRT and/or VMAT, in order to match the calculated dose to measurements [15]. To accurately calculate the dose distribution of the IMRT and VMAT, it is important to characterize the LTF and DLG. However, few studies have reported the inter-unit variability of these parameters, both before and after customization. If the variation of the parameters optimized for IMRT and VMAT are small, common parameter values may be usable for each treatment unit, resulting in shortened commissioning of the IMRT and VMAT. Here we performed a multi-institutional survey, among institutions using Varian linacs, to investigate the usage conditions of the IMRT/VMAT, measured parameter values and values registered to the TPS.

## MATERIALS AND METHODS

In this study, we conducted a survey using a web-based questionnaire using Google Forms (Google LLC, Mountain View, CA, USA). Sixty-nine sets of linac data were collected from 58 institutions. The linacs equipped the Millennium 120 MLC or HD120 MLC (Varian Medical Systems). Details of the MLCs are shown in Table 1. In Table 2, the number of linacs investigated in this study are listed with the type of MLC. C-Series linacs with the Millennium 120 MLC included Clinac iX, Trillogy and 6EX. All C-series linacs equipping the HD120 MLC were Novalis Tx (Varian Medical Systems and BrainLAB, Munich, Germany). For C-series linacs with the Millennium 120 MLC, the number of machines validating 4, 6 and 10 MV photons were 17, 16 and 32, respectively. For Novalis Tx, the number of units validating 6 and 10 MV photons was 9 and 10, respectively. All TrueBeam linacs validated 4, 6 and 10 MV flattened photon beams and 6 and 10 MV flattening filter-free (FFF) beams.

**Table 1:** Characteristics of MLCs

Indices		Millennium 120 MLC	HD120 MLC
Leaf thickness (cm)		6.7	6.9
Tip radius of curvature (cm)		8	16
Inner leaves	Number	40	32
	Width (cm)	0.5	0.25
Outer leaves	Number	20	28
	Width (cm)	1	0.5

**Table 2:** Number of treatment units analysed in this study

MLC	TrueBeam		C-series		Total	
Millennium120	18	(26%)	33	(48%)	51	(74%)
HD120	7	(10%)	11	(16%)	18	(26%)
Total	25	(36%)	44	(64%)	69	(100%)

In our survey, we collected (i) ionization chambers used for measurements of the MLC parameters, (ii) phantom used for measurements, (iii) source-to-surface distance (SSD) and measurement depth, (iv) beam sequence used for measurement of DLG and (v) LTF and DLG values. If the parameters registered to the TPS were customized, the methods and the values optimized for IMRT/VMAT were also collected.

For statistical analysis, JMP Software ver. 14.0 (SAS Institute, Cary, NC, USA) was used. For flattened beams, the MLC parameter values were grouped into four classes in accordance with the treatment machine and MLC, and the Steel-Dwass test was used. As only TrueBeam can generate FFF beams, parameter values of FFF beams were separated into two groups by the MLC, and the Wilcoxon signed rank test was used to analyse this data. Statistical significance was defined as a *P*-value < 0.05.

## RESULTS

All 58 institutions used ionization chambers to measure MLC parameters. Table 3 summarizes the conditions of the measurements. Farmer-type ionization chambers with a sensitive volume of 0.6 cm^3^ were used at 79% of institutions. Water phantom was used at 83% institutions. The most used geometry was SSD = 90 cm with 10 cm depth. Fifty-three institutions (91%) used a vendor-provided plan of sweeping gap irradiation for measurements of DLG, whereas five institutions (9%) used treatment plans that were generated in-house.

**Table 3:** Summary of the conditions for measurements

Category	Index	*n*	(%)
Ionization chamber	0.6 cm^3^	46	(79%)
	0.4 cm^3^	1	(2%)
	0.1 cm^3^	11	(19%)
Phantom	Water phantom	48	(83%)
	Solid phantom	10	(17%)
SSD/depth	100 cm/10 cm	2	(3%)
	90 cm/10 cm	52	(90%)
	Other	4	(7%)
Leaf motion for DLG	Vendor-provided plan	53	(91%)
	Other	5	(9%)

**Fig. 1:**
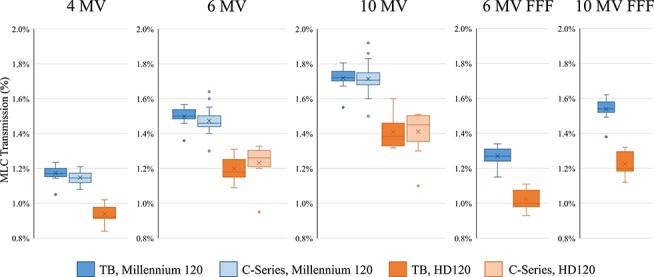
Box-Whisker plot of the MLC leaf transmission factor. TB = TrueBeam.

Table 4 summarizes the measured LTF values, and Fig. 1 shows the Box-Whisker plot of the LTF values. For both C-series and TrueBeam linacs, the HD120 MLCs showed significantly smaller transmission (*P* < 0.005). When comparing the LTF of linacs equipping the same MLC, there was no significant difference between the LTF of the TrueBeam and C-series for both Millennium 120 and HD120 MLCs.

**Fig. 2:**
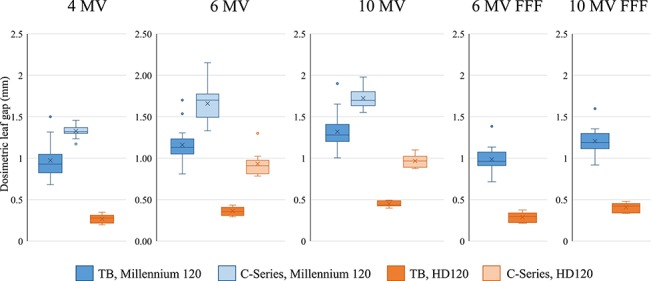
Box-Whisker plot of the dosimetric leaf gap. TB = TrueBeam.

Table 5 summarizes the measured DLG values. Figure 2 shows the box-whisker plot of the measured DLG values. For both C-series and TrueBeam linacs, the DLG values of the Millennium 120 were significantly larger than those of the HD120 MLCs (p < 0.001). When comparing the DLG of the linacs equipping the same MLC, the DLG values of the 6- and 10-MV flattened photon beams showed statistically significant differences between the TrueBeam and C-series for both Millennium 120 and HD120 MLCs (p < 0.005).

Among the institutions using TrueBeam linacs, 22 linacs (88%) used VMAT and eight of them (32%) used both IMRT and VMAT techniques. Thirteen institutions (68%) customized the MLC parameters registered to the Eclipse TPS. In contrast, for C-series linacs, 25 linacs (48%) used IMRT and 14 of them (32%) used both IMRT and VMAT. Only four institutions (9%) customized the MLC parameters registered to TPS.

**Table 4:** Mean ± SD (%) of MLC leaf transmission factors

Energy	Millennium 120		HD120	
	TrueBeam	C-Series	TrueBeam	C-Series
4 MV	1.17 ± 0.04	1.15 ± 0.04	0.94 ± 0.06	
6 MV	1.50 ± 0.05	1.47 ± 0.08	1.20 ± 0.08	1.23 ± 0.11
10 MV	1.72 ± 0.06	1.71 ± 0.07	1.41 ± 0.10	1.41 ± 0.13
6 MV FFF	1.27 ± 0.05		1.02 ± 0.06	
10 MV FFF	1.54 ± 0.06		1.23 ± 0.07	

Table 6 summarizes the number of institutions listed against the methods and criteria for determination of optimal MLC parameters. Note that the values represent the number of institutions but not the number of machines. Most institutions used clinical treatment plans for adjusting the MLC parameters. Ten institutions (58.8%) used ionization chambers, whereas the remaining seven institutions (41.2%) used 3D array detectors. For 3D array detectors, all facilities evaluated the gamma passing ratio, although inter-institutional variability of criteria was observed.

**Table 5:** Mean ± SD (mm) of dosimetric leaf gap values

Energy	Millennium 120		HD120	
	TrueBeam	C-Series	TrueBeam	C-Series
4 MV	0.97 ± 0.21	1.33 ± 0.07	0.27 ± 0.05	
6 MV	1.16 ± 0.22	1.66 ± 0.21	0.36 ± 0.05	0.93 ± 0.15
10 MV	1.32 ± 0.21	1.72 ± 0.12	0.45 ± 0.04	0.97 ± 0.08
6 MV FFF	0.99 ± 0.16		0.29 ± 0.06	
10 MV FFF	1.21 ± 0.16		0.41 ± 0.06	

**Fig. 3:**
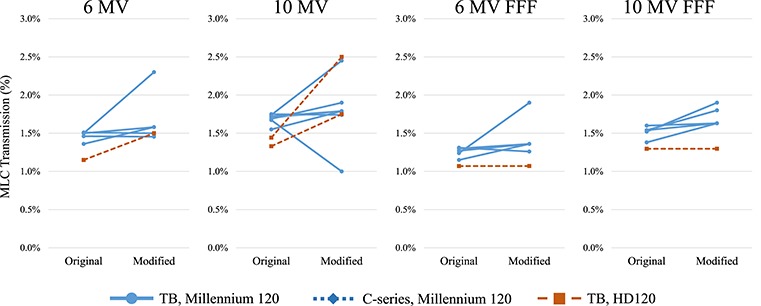
MLC transmission values before and after modification. TB = TrueBeam.

**Fig. 4:**
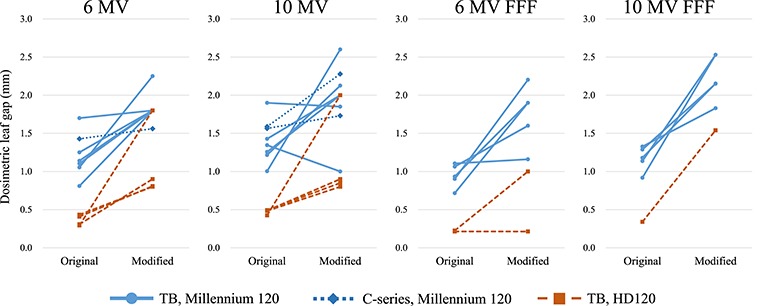
Dosimetric leaf gap values before and after modification. TB = TrueBeam.

Figure 3 shows the LTF values of institutions that modified the values for IMRT/VMAT planning. Because a 4 MV photon beam is not usually used for IMRT/VMAT treatments, very few institutions modified the LTF of 4 MV beams. Among all beams modifying the LTF, except the 4 MV, 82% of machines increased the values from the measurements. On average, the amount of adjustment, defined as (LTF_modified_/LTF_measured_ − 1) × 100%, was 0.17, 0.09, 0.16 and 0.17% for 6 MV, 10 MV, 6 MV FFF and 10 MV FFF beams, respectively. Figure 4 shows the DLG values of the institutions that modified the values for IMRT/VMAT planning. Among all beams modifying the DLG, except the 4 MV, 92% of machines increased the DLG values. On average, the amount of the adjustment, calculated as the DLG_modified_–DLG_measured_ was 0.60, 0.57, 0.81 and 1.07 mm for 6 MV, 10 MV, 6 MV FFF and 10 MV FFF beams, respectively. Table 7 shows the median and 95% confidence interval of the modified LTF and DLG values for TrueBeam equipping the Millennium 120 MLC. Machines adjusting either LTF or DLG were included.

## DISCUSSION

In this study, we collected the MLC parameters from multiple institutions using Varian linacs and investigated their inter-unit variability. The difference in MLC transmission is caused by the difference in thickness between the Millennium120 MLC (6.7 cm) and the HD120 MLC (6.9 cm). The HD120 MLC showed significantly smaller DLG values, probably because the radius of the curvature of the HD120 MLC leaf end is 16 cm, which is larger than that of the Millennium 120 MLC (8 cm) [15]. Interestingly, the TrueBeam and C-series linacs showed significant differences of the DLG values even with the same model of MLC. According to the vendor, such variations were likely due to the method of setting the MLC leaf origin during machine installation. For TrueBeam linacs, the origin of MLC leaves are adjusted at the isocenter based on the light field. In contrast, the MLC origin of the C-series is adjusted at the level of the linac head by attaching an alignment bar to the linac head. This difference will cause the systematic difference in the DLG values between the two linac models.

A few studies have reported the LTF and DLG values of TrueBeam linacs. Chang *et al*. compared the beam data of three TrueBeam machines and reported the leakage to be 7–17% lower than this study, as well as DLG values 30–42% lower than we found [6]. Glide-Hurst *et al*. collected beam data of five TrueBeam machines and reported LTF values 4–7% larger than this study, as well as DLG values 13–24% larger than we found [4]. These differences may be due to unstandardized measurement protocols including the ionization chambers, SSD, measurement depth and plan sequences. According to the vendor-provided reference guide, the effective leaf transmission values are affected by the measurement devices, and they are also slightly affected by the field size and measurement depth [12].

**Table 6:** Methods and criteria for determination of optimal MLC parameters for IMRT and VMAT

Category	Index	*n*
Plan	Clinical plan	16
	Phantom plan	1
Number of cases	<5	6
	5–9	4
	10–14	4
	15–19	1
	≥20	2
Ionization chamber (*n* = 10)		
Criteria (point dose)	1%	2
	2%	6
	3%	2
3D array detector (*n* = 7)		
Criteria (gamma pass rate)	90%	2
	95%	4
	98%	1
Gamma thresholds (DD/DTA)	2%/2 mm	2
	3%/2 mm	4
	3%/3 mm	1

DD = dose difference, DTA = distance-to-agreement.

In this study, the coefficient of variation (CV), calculated as the standard deviation (SD) divided by the mean value, was within 6 and 10% for the transmission of Millennium 120 and HD120 MLCs, respectively, whereas the DLG showed larger CV, exceeding 20%. As shown in Table 2, five institutions (9%) used other techniques to measure DLG. This may lead to larger inter-institutional variations for DLG than for LTF.

The LTF and DLG values registered to the TPS greatly affect the dose calculations of the IMRT and VMAT. However, the measured values are often not appropriate. Kielar *et al*. reported that use of DLG derived from a sweeping test for VMAT calculations resulted in differences >5% between the calculations and measurements [15]. In such cases, the DLG and/or LTF values are iteratively changed until the optimal values are identified. In addition, single DLG values cannot be appropriate for all treatment plans with various field sizes [13, 16], although only one DLG value can be registered to the Eclipse TPS. Wen *et al*. measured the LTF and DLG using vendor-recommended protocols and optimized the values based on the measurements of the American Association of Physicists in Medicine (AAPM) Task-Group 119 [17] RapidArc plan using an ionization chamber [18]. They adjusted the DLG values by 0.25-0.70 mm from the measured values, whereas the change in the LTF was modest. In this study, the amount of DLG and LTF adjustments were on average 0.6–1.1 mm and 0.09–0.17%, respectively.

The MLC parameters optimized for IMRT/VMAT calculations showed inter-institutional variations. As demonstrated in Table 6, the methods and criteria for adjustments of MLC parameters also showed variability among the institutions. This may lead to the variability of the optimized values. However, there appeared to be a few common trends such as the DLG values were upwardly changed and LTF values were adjusted modestly. A few studies also reported similar adjustments of the MLC parameters [18, 19]. Although the MLC parameters shown in our current study will not be optimal for all treatment machines, these values can be used as a starting point for the iterative processes to find the optimal values.

**Table 7:** Median and 95% confidence interval (CI) of the modified MLC leaf transmission factors and dosimetric leaf gap values for TrueBeam equipping the Millennium120 MLC

Parameter	6 MV		10 MV		6 MV FFF		10 MV FFF	
	Median	(95% CI)	Median	(95% CI)	Median	(95% CI)	Median	(95% CI)
Transmission	1.65%	(1.38–1.92)	1.78%	(1.39–2.17)	1.41%	(1.16–1.67)	1.68%	(1.53–1.83)
DLG (mm)	1.78	(1.49–2.06)	1.93	(1.38–2.48)	1.75	(1.26–2.24)	2.24	(1.87–2.61)

This study, however, included the following limitations: (i) protocols of the measurements and fine-tuning of the MLC parameters were not standardized, and (ii) we did not evaluate the dose calculation accuracy of the IMRT/VMAT for each treatment unit. As mentioned above, MLC parameters are affected by the measurement devices and conditions. In addition, the optimal values depend on treatment plan characteristics generated by each institution, such as the field size and complexity of the treatment plans. The optimal MLC parameters should be carefully determined according to each institution.

We evaluated the MLC parameters, including the LTF and DLG, collected from multiple institutions. Many of the institutions studied used common measurement protocols, although variabilities were also observed. For optimization of the parameters for IMRT/VMAT calculations, DLG values were upwardly adjusted at many institutions, whereas the LTF values were modestly changed. Although fine-tuning of the MLC parameters will be needed at each institution, use of the mean values obtained in this study may be helpful to shorten the iterative processes of finding the optimal values.
